# Determinations of Melamine Residue in Infant Formula Brands Available in Iran Market Using by HPLC Method

**Published:** 2018

**Authors:** Javad Maleki, Firouzeh Nazari, Jamal Yousefi, Roya Khosrokhavar, Mir-Jamal Hosseini

**Affiliations:** a *Department of Food Safety and Hygiene, School of Public Health, Zanjan University of Medical Sciences, Zanjan, Iran. *; b *Food and Drug Administration, Iran University of Medical Sciences, Tehran, Iran. *; c *Food and Drug Laboratory Research Center, Ministry of Health and Medical Education, Tehran, Iran.*; d *Zanjan* * Applied Pharmacology Research Center, Zanjan University of Medical Sciences, Zanjan, Iran. *; e *Department of Pharmacology and Toxicology, School of Pharmacy, Zanjan University of Medical Sciences, Zanjan, Iran.*; 1 *J. M. and F. N. contributed equally to this work.*

**Keywords:** Melamine, Infant formula, Follow up formula, Exposure assessment, HPLC

## Abstract

The contamination of melamine was evaluated in 69 infants along with follow up formula samples collected from the market for the first time in Iran using HPLC method. Since there are no previous data concerning the contamination level of melamine in all brands of infant formula samples consumed using the HPLC method in Iran, this study is the first investigation in this regard. Our results showed that melamine contamination was found in 65% of samples, where mean and maximum levels of melamine were 0.73 ± 0.71 mg/kg and 3.63 mg/kg, respectively. The level of melamine in 10 out of 69 samples was higher than the maximum level set by the Codex Alimentarius in infant food (1 mg/kg). Melamine was determined in 67.8% and 50% of domestic and imported samples, respectively. The estimated daily intake was designed in two scenarios: it was calculated based on the mean level of melamine contamination and maximum level of melamine in the samples. In both scenarios, our results showed that melamine intake across all age groups is lower than the tolerable daily intake (TDI) of 0.2 mg/kg body weight, suggested by WHO (0.2 mg/kg body weight). Thus, it seems that the current levels of melamine in infant and follow up formula purchased in Iran pose no health risk for infants.

## Introduction

Melamine (1, 3, 5-triazine-2, 4, 6-triamine) is a nitrogen-rich industrial chemical which is used in a variety of materials, including plastics, insulation, cleansers, and flame retardants (1, 2). Since melamine is a nitrogen-rich compound, it is used illegally in food and/or feed commodities to increase dummy protein content (3, 4). General attention to melamine began in 2007 when a large number of pet deaths occurred due to kidney failure, and scientists observed melamine contamination in pet food (5). In 2008, thousands of Chinese infants and young children were hospitalized for urinary problems due to the consumption of melamine-contaminated infant formula and related dairy products (6). In addition to infant formula, melamine-contaminated milk as ingredients has been found in liquid milk, frozen yogurt dessert, powdered milk, cereal products, canned coffee drink, protein powders confectionaries, cakes and biscuits, and some processed foodstuff (7). Available publications report that the most commonly observed toxic effects in animal experiments include a variety of toxic effects, including reduced food consumption, body weight loss, bladder stones, crystalluria, epithelial hyperplasia of urinary bladder, lowered survival rate, chronic kidney inflammation, and bladder carcinoma (3, 4 and 8). The number of articles published on melamine adulteration suggests renal complications in numerous cases especially in children and adults (9-11). The outbreak of urinary stones in children younger than 3 years in China suggested consumption of melamine in food, especially dairy products after approximately 3-6 months of exposure (12).

After finding melamine in foods or feed imported from China in 2007, many countries have raised widespread food safety concerns to minimize and control food compounds to preclude its adverse effects (5-7, 12 and 13). The maximum residual level of melamine content in infant formula has been legally regulated at 1 mg/kg by the Food and Drug Administration (FDA) and World Health Organization (WHO) (14-16). A new tolerable daily intake for melamine was established at 0.2 mg/kg body weight by the WHO in 2008 (15). 

Based on the previous studies, high-performance liquid chromatography (HPLC) has been approved as the gold standard and powerful technique for the analysis of melamine in biological, environmental, and food materials due to its high sensitivity, high specificity, shortest detection time, and low cost compared to the other methods (1, 17-20). 

Melamine contamination in milk product and infant formula has been reported in some studies. Deabes and El-Habib (21) evaluated melamine content in 22 samples including infant milk formula, growth milk formula, and full cream milk powder. Melamine was found in 100% of the analyzed samples, and the highest melamine content of 258 mg/kg was found in infant milk formula. In another study, in Dar-Es-Salaam (Tanzania, East Africa), melamine was found in 6% (3 of 49) of the analyzed milk powder and infant formula samples. The highest concentration of melamine (5.5 mg/kg) was seen in the milk powder samples (2).

**Table 1:** Results of method for determination of melamine in infant formula samples (n = 3).

**Spike level (mg/kg)**	**Recovery%**	**RSD%**
1	101.3	4.4
2.5	95.6	5.9
5	106.9	5.7

**Table 2:** Melamine content in the infant and follow up formula samples

**Sample Code**	**Melamine Concentration** **(mg/kg)**	**Sample Code**	**Melamine Concentration** **(mg/kg)**	**Sample Code**	**Melamine Concentration (mg/kg)**
1	0.32	24	ND	47	0.11
2	0.58	25	ND	48	0.22
3	0.64	26	0.55	49	ND
4	ND	27	0.12	50	0.46
5	0.86	28	ND	51	ND
6	1.48	29	ND	52	0.11
7	1.37	30	ND	53	0.12
8	0.33	31	0.49	54	0.60
9	0.70	32	ND	55	0.48
10	0.28	32	0.22	56	0.28
11	ND	34	ND	57	1.13
12	0.73	35	0.65	58	0.31
13	0.55	36	0.13	59	0.49
14	1.38	37	1.66	60	0.18
15	2.48	38	0.72	61	0.82
16	ND	39	ND	62	0.13
17	ND	40	ND	63	14.91
18	ND	41	ND	64	0.11
19	0.13	42	0.49	65	0.22
20	ND	43	ND	66	ND
21	ND	44	0.22	67	0.46
22	ND	45	ND	68	ND
23	ND	46	0.65	69	0.11

ND: non detected.

**Table 3:** Occurrence of melamine for groups in the analyzed samples

**Origin**	**Samples**	**Mean (mg/kg)**	**Range** **(mg/kg)**	**No. Of positive samples (%)**	**No. Of sample**
Domestic	Infant formula	0.78	0.11-3.63	30 (71.4)	42
	Follow up formula	0.72	0.12-2.48	10 (58.8)	17
Imported	Infant formula	0.46	0.25-0.65	3 (50)	6
	Follow up formula	0.38	0.38	2 (50)	4
Total	Infant formula	0.75	0.11-3.63	33 (68.8)	48
	Follow up formula	0.68	0.12-2.48	12 (57.2)	21

**Table 4:** The range of melamine in the infant and follow up formula samples

**Range (mg/kg)**	**No. of samples**	**Contamination (%)**
ND*	24	34.8%
0.1 < X <1	35	50.7%
1 ≤ X	10	14.5%

LOD = 0.03 mg/kg; LOQ were 0.1 mg/kg.

* ND: Not detected ≤ LOQ (0.1 mg/kg).

**Table 5:** Natural occurrence of melamine in infant and follow up formula obtained from available brands.

**Producer**	**Mean ** **±** ** SD (mg/kg)**
A	0.96 ± 0.64
B	0
C	0.25
D	0.56 ± 0.61
E	0.55 ± 0.59
F	0.78 ± 0.95
G	0
H	0
I	0
J	0
K	0.52 ± 0.19

**Table 6:** Daily intake of melamine from infant and follow up formula consumption

**Age (month)**	**Consumption (kg/month)**	**Average of body weight (kg)**	**Daily intake (mg/kg b.w/day)**	
			**Mean**	**Max**
<6	1.6-3.2	5.7	0.007-0.0135	0.034-0.068
7-8 month	1.2-2.4	6.9	0.004-0.008	0.014-0.029
9-12 month	0.8-1.6	9.5	0.002-0.004	0.007-0.014

**Figure 1:**
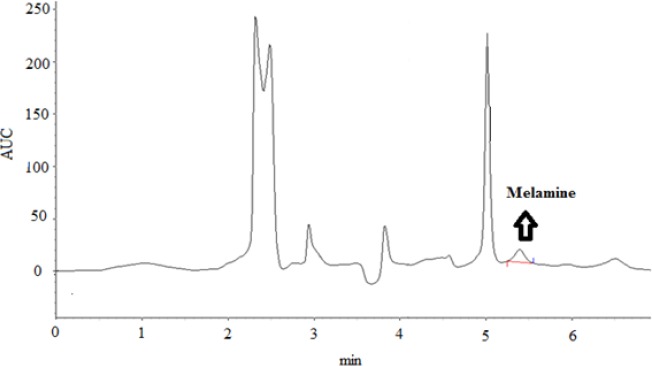
HPLC chromatograms of an infant formula with melamine content of 0.39 mg/kg.

There are little data on melamine contamination in food in Iran investigating the occurrence of melamine in nine powder milk and five liquid milk samples from different brands available in Iran (22). Only one brand of milk powder and liquid milk showed no melamine contamination in determined melamine level in milk, yoghurt, infant formula, coffee mate and cheese samples collected in Zanjan, Iran (22). They reported the mean values of melamine in milk, yoghurt, infant formula, coffee mate, and cheese as 0.24, 0.76, 1.38, 0.56 and 1.16 mg/kg, respectively. 

 As China is the major supplier of infant and follow up formula materials for Iran, the objective of this study was to evaluate the presence and level of melamine in all available brands of infant and follow up formula in Iran using SPE column and HPLC analysis.

## Experimental

*Samples*

A total of 48 infant formulas (from birth to 6 month) and 21 follow up formula (from birth to 12 month) samples from 11 different brands (5 domestic brands and 6 imported) were collected randomly from drugstores in Zanjan, Iran, during April to November. The samples were stored at room temperature until analysis.

*Chemicals*

Melamine (99.0% purity) and Sodium 1-octane sulfate (98%), were purchased from Sigma-Aldrich (St. Louis, MO). Acetonitrile (ACN) and methanol (both HPLC grade), citric acid, ammonia solution (25–28%), hydrochloric acid fuming 37% (HCL) and sodium hydroxide (NaOH), and trichloroacetic acid (TCA) were obtained from Merck, Darmstardt, Germany). Ultra-pure water was produced from distilled water by a Water Purification system (Q-cheek, USA).

*Standard, sample and solvent preparation*

A 1000 μg/mL melamine stock standard was prepared by accurately weighting 100 mg of melamine into a 100 mL volumetric flask. Melamine was dissolved with deionized water with aid of sonication and heating for 15 min. The stock standard was diluted appropriately to prepare working standards with concentration ranging from 0.1-10 μg/mL to obtain a calibration curve.

*HPLC parameters*

Detection and quantification were performed using an HPLC system (KNAUER, Berlin, Germany) equipped with an isocratic pump Model 1000 (KNAUER, Smartline, Germany) and UV detector Model 2600 (KNAUER, Smartline, Germany). Instrument control and data acquisition were performed with a personal computer running the open LAB chromatography data systems (CDC) EZchrom Elite (KNAUER, Smartline, Germany). Chromatographic separation was performed at 30 °C on a Eurosphere (RP-C18, 250 × 4.6 mm ID, 5 μm, KNAUER, Berlin, Germany) and a mobile phase consisted of 10 mM of Sodium 1-octane sulfate, 10 mM of citric acid buffer and acetonitrile (ACN) (80:20 v/v, pH 3.0) with a ﬂow rate of 1.0 mL/min and melamine was detected at 244 nm. 

*Methodology*

The extraction and cleanup procedures were carried out by using Hassani *et al.*, 2013. Fifteen mL of TCA (1% v/v) and 5 mL of ACN were added to 2 g of an infant or formula sample in a 50 mL centrifuge tube. After 1 min of vortex and shaking and 30 min of ultra-sonication, the sample was centrifuged for 10 min (10000 rpm). The supernatant was diluted with TCA (1% v/v) to reach in 25 mL. Clean up sample was done by using a solid phase extraction (SPE) CHROMABOND® HR-XC polypropylene column (85 μm/mL/200 mg). The SPE column was initially activated by passing 5 mL of CH3OH and 5 mL of H2O. After sample addition to the SPE column, the column was eluted with 6 mL of ammoniated methanol solution (mixture of 5 mL of ammonia solution and 95 mL of methanol). Then, the collected sample was dried under a gentle stream of nitrogen gas and dissolved in 1 mL of mobile phase and filtered prior to injection. The accuracy and precision of the method were determined by spiking an infant formula sample with different concentrations of melamine (1, 2.5, and 5 mg/kg, n = 3 replicates).

*Statistical analysis*

All statistical analyses were performed using the SPSS software (Window version 18) and Excel 2007 software were used for data analysis. The probability value of *p* < 0.05 was considered as statistically significant in this research.

## Results and Discussion

Melamine was quantified in infant and follow up formula samples (n = 69) using external calibration curve. Based on the validation method, our results confirm that our method is suitable in terms of reliability, reproducibility, and usefulness for routine monitoring of melamine in infant and follow up formula. The accuracy of the method was checked using the recovery test. Limit of quantification (LOQ) and limit of detection (LOD) were estimated at the lowest concentration in spiked samples corresponding to a signal-to-noise ratio (S/N) of 10:1 and 3:1, respectively. 

The linearity was prepared from stock solution at seven concentration levels ranging from 0.1 to 10 μg/mL. Triplicate injections were performed randomly at each level. One-hundred microliter of the prepared samples or standard solutions was directly injected into the HPLC system. A calibration curve was obtained by plotting the peak area and concentration.

The method performance characteristics of the analytical method established are shown in Table 1. LOD and LOQ were 0.03 and 0.1 mg/kg, respectively. The recoveries reflected a three-fold average of 3 analyses at each concentration, as shown in Table 1. The accuracy of the method was assessed by performing recovery experiments in three spiked levels within an acceptable range with permissible RSD%. To evaluate the reliability of the results, a blank and a spiked sample were also analyzed in each working day. The retention time of melamine in the standard and spiked samples of melamine was 5.30 ± 0.1 min (Figure 1).

Melamine content in the analyzed infant and follow up formula samples has been indicated in Table 2. According to the results, melamine was found in 71.7%, 58.8%, and 50% of domestic infant formula, domestic follow up formula and imported follow up formula, respectively. The mean and maximum levels of melamine in positive samples were 0.73 ± 0.71 and 3.63 mg/kg, respectively. The maximum level of melamine contamination was observed in the domestic infant formula with the results shown in Table 3. The range of melamine contamination in the samples was shown in Table 4.

Our results revealed that melamine was found in 65.2% of infant formula samples and melamine level in only 14.5% of samples was observed to be higher than the maximum level set by the Codex Alimentarius for infant food (1 mg/kg). The low level of melamine has probably come from a different source. Since melamine is a raw material in the production of some plastic products used for serving food, foods may contain low melamine content because of its migration into the food or degradation of some chemicals to melamine (15). 

This is the first study which investigates the melamine level across all infant and follow up formula brands available in Iran. Unfortunately, the Institute of Standards and Industrial Research of Iran has no regulations regarding melamine contamination in food products. Consequently, these results were compared with the maximum residual levels specified by some other countries. There are little data on the melamine contamination in infant and follow up formula in Iran. 

According to a study in Iran, melamine was found in 96% of infant formula samples (n = 15) within the range of 0.35–3.40 mg/kg with a mean of 1.49 ± 1.25 mg/kg. In 4 out of 15 samples (26.6%), the level of melamine was higher than the maximum level established by the Codex Alimentarius in infant food (1 mg/kg) (22). This study was in agreement with our results confirming that the content of melamine in most of the samples is lower than the respective maximum level. Considering the data from other countries, (23) 94 analyzed samples of infant formula were purchased in Canada. Melamine was detected in 71 of the 94 samples at concentrations ranging from 4.31 to 346 μg/kg (median = 16 μg/kg). Different brand analysis can be one of the reasons for incongruence in our results with the previous study. Further, even a different batch number form similar brands could mean different amounts of melamine. Also, some factories produce different products from developed and other countries.

In this study, melamine concentration was not above the level recommended by the Codex Alimentarius in infant foods in any of the samples. Another study evaluated the existence of melamine in infant formula samples collected in Dar-es-Salaam (183 Tanzania, East Africa) and reported that a total of 6% (3 of 49) of analyzed samples were contaminated by melamine within the range of 0.5 and 5.5 mg/kg (2). The Kruskal–Wallis test, which is based on nonparametric tests, showed that there was a significant difference in terms of levels of melamine across different brands of samples. (Table 5).

*Estimated daily intake*

In order to evaluate the potential health risk of melamine, daily melamine intake from the consumption of milk powder was calculated. According to WHO guideline, TDI for melamine is 0.2 mg/kg body weight for all ages of the population, including infants. The intake of melamine depends on melamine concentration in milk powder and the amount of milk powder daily consumed. 

The data of infant formula consumption and children body weight are based on the reports by World Health Organization (24). Hence, there are no separate data of infant and follow up formula consumption; thus, the mean of total samples was used for estimation of melamine daily intake. The average infant and follow up formula consumption in Iran for different age groups and melamine intake are shown in Table 6. In this study, the daily intake was designed in two scenarios: it was calculated based on the mean level of melamine contamination and the maximum level of melamine in the samples. As shown in Table 5, melamine intake across all age groups was lower than the tolerable daily intake set by WHO 2009, suggesting no health risk of consumers according to the obtained data (15). However, 10 samples of infant and follow up formula had a higher than the level of melamine maximum level set by the Codex Alimentarius in infant food (1 mg/kg). We believe that a thorough study is required to determine the melamine concentration in infant and follow up formula in Iranian and imported products for continuous monitoring. 

Also, some rules need to be executed in this regard to minimize the contamination to the least possible amount and to preclude its adverse effect. More studies are needed to properly assess other sources and to compare with other methods for estimation of melamine in infant formula and dairy 

product.

## Conclusion 

Since there are no previous data concerning the contamination level of melamine across all brands of infant formula samples consumed using HPLC method in Iran, this study is the first investigation to confirm the existence of melamine in infant formula in the market, and to provide a basis for future studies. In this study, an accurate, simple, sensitive, reproducible, and minor modified analytical method are reported for the determination of melamine in infant and follow up formula. The suggested method requires short times with flexible and suitable for the validation of minor suggested method. This study involved optimization of HPLC-UV with satisfied results in terms of linear range, LOD, LOQ, and reproducibility using a spiked sample. The results suggest that Iranian children fed on infant and the follow up formula are exposed to various levels of melamine. Although the high incidence of melamine contamination was found in the analyzed samples, daily intake of melamine by different age groups of children was lower than TDI set by WHO suggesting that the presence of these levels of melamine in infant and follow up formula collected in Iran does not pose a health risk.
